# Aetiology of Pulmonary Symptoms in HIV-Infected Smear Negative Recurrent PTB Suspects in Kampala, Uganda: A Cross-Sectional Study

**DOI:** 10.1371/journal.pone.0082257

**Published:** 2013-12-03

**Authors:** Alphonse Okwera, Freddie Bwanga, Irene Najjingo, Yusuf Mulumba, David K. Mafigiri, Christopher C. Whalen, Moses L. Joloba

**Affiliations:** 1 Department of Internal Medicine, School of Medicine, Makerere University College of Health Sciences, Kampala, Uganda; 2 Department of Medical Microbiology, School of Biomedical Sciences, Makerere University College of Health Sciences, Kampala, Uganda; 3 Uganda-Case Research Collaboration Kampala, Uganda; 4 School of Social Sciences, Makerere University College of Humanities and Social Sciences, Kampala, Uganda; 5 Department of Epidemiology and Biostatistics, School of Public Health, University of Georgia Atlanta, United States of America; University of Cape Town, South Africa

## Abstract

**Introduction:**

Previously treated TB patients with pulmonary symptoms are often considered recurrent TB suspects in the resource-limited settings, where investigations are limited to microscopy and chest x-ray. Category II anti-TB drugs may be inappropriate and may expose patients to pill burden, drug toxicities and drug-drug interactions.

**Objective:**

To determine the causes of pulmonary symptoms in HIV-infected smear negative recurrent pulmonary tuberculosis suspects at Mulago Hospital, Kampala.

**Methods:**

Between March 2008 and December 2011, induced sputum samples of 178 consented HIV-infected smear negative recurrent TB suspects in Kampala were subjected to MGIT and LJ cultures for mycobacteria at TB Reference Laboratory, Kampala. Processed sputum samples were also tested by PCR to detect 18S rRNA gene of *P.jirovecii* and cultured for other bacteria.

**Results:**

Bacteria, *M. tuberculosis* and *Pneumocystis jirovecii* were detected in 27%, 18% and 6.7% of patients respectively and 53.4% of the specimens had no microorganisms. *S. pneumoniae*, *M. catarrhalis* and *H. influenzae* were 100% susceptible to chloramphenicol and erythromycin but co-trimoxazole resistant.

**Conclusion:**

At least 81.5% of participants had no microbiologically-confirmed TB. However our findings call for thorough investigation of HIV-infected smear negative recurrent TB suspects to guide cost effective treatment.

## Introduction

Pulmonary infection is the leading cause of morbidity and mortality in HIV-infected individuals [1,2,3,4,5,6]. The clinical presentation of HIV/AIDS and the occurrence of opportunistic infections depend on different factors such as the presence of endemic diseases, quality of health services, availability of and access to anti-retroviral treatment and levels of education of the population [7]. Recent studies in Uganda [8,9,10,11] and in sub-Saharan Africa [12,13] have demonstrated that 9% to 38.6% of HIV infected patients with negative AFB sputum smear and pulmonary infiltrates that do not respond to antibiotics were in fact *Pnuemocystis jirovecii* (PCP) pneumonia. Worodria’s study in particular found 38.6% PCP, 24.1% TB and 8.4% bacteria in 83 AFB sputum smear negative HIV-infected patients with pulmonary symptoms [8]. However, Harris et al in Malawi confirmed pulmonary tuberculosis (PTB) bacteriologically in 42% of 60 smear negative PTB patients who had previously been treated [14]. The mortality associated with PCP among these individuals is high [15]. At the national tuberculosis treatment centre of the national tuberculosis programme, 30% of individual adults being evaluated for recurrent PTB in 2003-2007 were smear negative of whom 80% were HIV positive. There was high mortality despite initiation of anti-tuberculosis chemotherapy [16]. The aetiologies of pulmonary morbidity and mortality were not known as no further studies including cultures and antibiotic susceptibility testing were done or post mortem examinations performed on those who had died. Previously-treated TB patients who present with pulmonary symptoms are often suspected to have recurrent PTB. In the resource-limited settings, where routine investigations of such patients are limited to acid fast microscopy and chest x-ray, a definitive diagnosis of the aetiological agents is not usually made in those who remain smear negative. Most of such patients are treated blindly with category II anti-TB drugs. Category II TB treatment may be inappropriate and may expose patients to high pill burden, overlapping drug toxicities and drug-drug interactions. It is therefore prudent that a microbiological investigation be done in HIV-infected individuals with pulmonary infections to improve on the cost effective empirical treatment focusing on locally prevailing causative micro-organisms in resource limited settings. 

The goal of this study was to determine the prevalence of *P. jirovecii* and bacteria in smear negative HIV-infected adults being evaluated for recurrent pulmonary tuberculosis. Induced sputum samples were cultured for *M. tuberculosis* and other microorganisms and antibiotic susceptibility testing of the microorganisms isolated and assessment of CD4 cell counts were performed. 

## Materials and Methods

### Study population

This was a cross-sectional study which involved patients who came to attend the TB clinic. The case definition in this study was: An HIV infected adult who was previously treated for PTB and presents with a cough for two or more weeks with or without fever, chest pain, haemoptysis or dyspnoea, regardless of a recent antibiotic use except co-trimoxazole. Participants were screened by history, sputum smear and HIV testing and consecutively enrolled from March 2008 through March 2011 at the national TB treatment centre, Mulago Hospital-Kampala. Participants included adults aged 18-60 years, previously treated successfully for PTB presenting with cough symptoms of two or more weeks, either with or without fever, dyspnoea and chest pain. Other inclusion criteria were sputum smear negative, gave informed consent for HIV and tested positive, gave written informed consent to both undergo sputum induction and participate in the study. Participants were excluded from the study if they had other serious medical conditions such as cardiac disease, severe bronchial asthma, or mental illness and Karnofsky performance below 50%. These patients were referred from the medical wards, The AIDS Support Organization, Mulago–Mbarara Joint AIDS Programme and other TB diagnostic testing units in Kampala.

### Data collection

A pre-tested questionnaire was used to record socio-demographic and clinical data from the study patients. Investigations conducted included laboratory procedures, sputum induction and chest x-ray. Karnofsky performance status score was performed on all study patients. HIV staging was done using the WHO criteria [17]. Previous or current use of HAART and co-trimoxazole prophylaxis by the study patients were recorded as the use of HAART and co-trimoxazole may affect the yield of *P. jirovecii* and bacterial cultures. Study participants found to have *P. jirovecii* pneumonia were started on treatment.

#### Laboratory procedures

For each study patient, a complete blood count and haemoglobin were performed using Coulter counter machine. HIV status was determined using three rapid tests [18] as recommended by WHO and Uganda National Aids Programme [19] and CD_4_ and CD_8_ were determined by auto-software flow cytometer FACSCalibur^TM^ (BD Biosciences Becton, Dickinson and Company, San Jose, CA) at Mulago-Mbarara Joint AIDS Programme laboratory.

#### Sputum specimens

ZN staining and examination for AFB [20] were done on spot and early morning sputum specimens at the side laboratory of TB ward on all patients being screened for the study and thereafter study participants underwent sputum induction for microbiology studies.

#### Sputum induction procedure

The study patients took nothing by mouth for 6 hours to 8 hours prior to sputum induction. Before sputum induction, study patients cleansed the oral cavity (rinsed and gargled) with normal saline to remove food particles, and epithelial cells that might interfere with microscopic examination. The sputum induction room was well ventilated and equipped with adrenaline, hydrocortisone, oxygen, intravenous fluids and salbutamol inhaler for emergency use. Sputum was induced by inhalation of hypertonic saline (3%) generated by an ultrasonic hand held nebulizer (SUNRISE Somerset, PA 15501-2125 USA). The induction was continued for 15 to 20 minutes or until a specimen volume of 5–10 ml was produced. Minor adverse event associated with sputum induction was limited to coughing that caused vomiting in two participants. A trained nurse supervised this procedure: As a quality control measure, good quality of the induced sputum was ensured by assessing the number of epithelial cells and polymorph nuclear cells (PMN) in order to differentiate it from saliva or oropharyngeal secretion on Gram stain as follows: <1 epithelial cell or PMN = 1+, 1-10 = 2+, 11-25 = 3+ and >25 = 4+ (many) (CML-SP 503 version 1.0). Induced sputum containing many or numerous epithelial/PMN cells were considered saliva and unsuitable for the study.

#### Sputum culture for *M. tuberculosis*

 Induced sputum specimens from 2-8°C refrigerator were mucolysed by adding equal volume of reconstituted sputulysin to each sample. Ten millilitres of N-acetyl L-cysteine (NALC)-4% Sodium Hydroxide (NaOH) were added to digest and decontaminate the sputum specimens. The sputum specimens were diluted with sterile phosphate buffer, pH 6.8. The mixtures were then centrifuged at 3000 x g for 15 min and the sediments re-suspended in 1 to 2 ml of sterile phosphate buffer, pH 6.8. Ziel-Neelsen smears were made from the suspend sediment [20] and 2.5 ml inoculated in Lowenstein Jensen (LJ) media and Mycobacteria Growth Indicator Tube (MGIT) all from Becton and Dickson, Franklin Lakes, NJ USA at Uganda National Reference Laboratory, Wandegeya-Kampala [21,22]. Care was taken at each step to minimise generating aerosols. All samples that turned positive were subjected to ZN staining for AFB and blood agar to rule out contamination with other bacteria. Those which were positive were identified using Capilia TB Neo™ (TAUN, Numazu, Japan) assay for *Mycobacterium tuberculosis* complex and non tuberculosis mycobacterium. All results were entered into access-based laboratory data base for reporting.

#### *Pneumocystis jirovecii* DNA detection

Stored mucolysed induced sputum sample at -20°C was thawed to room temperature and vortexed gently. To extract *P. jirovecii* DNA, 20µL of induced sputum was added to 40µL of gene releaser assay as recommended by the manufacturer (Bio Ventures Inc., Murfreesboro, Tenn.). The induced sputum and gene releaser assay mixture was then heated at 100°C for 10 minutes and the cooled mixture was centrifuged at 1400 revolutions per minute for one minute. The supernatants which contained *P. jirovecii* DNA were then aliquoted, amplified in a thermo cycler and then subjected to agarose gel electrophoresis.

#### Primers and primer dilution

The primers (forward) PC41 (5`-CGAGACCTTAACCTACTAAATAGCCAGATT-3`) (Reverse) PC`22 (5`- AATGACCAAATTTGATCAACTTTCCAGCAA-3`) were diluted as shown in Table 1. Ten micro litres of the stock was added to 90µL of PCR water to make the working solution which was in the ratio 1 in 10.

**Table 1:** Primers and primer dilution for PCR to detect the 18S rRNA gene in *P.jirovecii* of HIV-infected smear negative recurrent PTB suspects, Kampala.

**Original conc. in mg**	**Conc. / µg**	**PCR water added (1µg/µl)**
Pc 41-0.31	310	310
Pc 22-0.29	290	290

#### PCR Amplification

After DNA extraction,11µL reagent mixture containing: 8µL of PCR water,1µL of each of the primers,1µL of master mix and 0.2µL of Taq DNA pol was added into the PCR reaction tube. 3µL of the processed samples as DNA template were then added. The primers PC41 and PC22 were used to amplify a 418-bp fragment of the target gene. After an initial 5 minutes denaturation step at 95°C, DNA was amplified for 43 cycles with a final extension period of 10 minutes at 72°C. Each cycle consisted: 1 minute of denaturation at 94°C, 1 minute of extension of annealing at 56°C, and 1 minute of extension at 72°C.

#### Agarose gel electrophoresis

The amplified product was subjected to agarose electrophoresis. One and half grams of agarose was weighed and dissolved in 150ml of TBE buffer. The solution was boiled in a microwave for 2 minutes and the allowed to cool at 50°C before addition of ethidium bromide. The agarose poured on and allowed to set on the electrophoresis apparatus. Four microlitres of loading dye were added to 9 µL the amplified DNA sample and loaded on the wells. The samples were run at 100 volts for 60 minutes and visualized for expected *P. jirovecii* specific bands consisting of 418bp fragments after ethidium bromide staining. 

#### Sputum culture for bacteria

The most purulent part of induced sputum samples were selected using an inoculating loop and inoculated on plates of 5% sheep blood agar, chocolate agar, MacConkey agar for bacteria culture and Sabouraud`s dextrose agar for fungi. The blood agar and chocolate agar plates were inoculated at 35°-37°C under carbon dioxide while MaConkey agar plates and Sabourauds dextrose agar were inoculated at 35°-37°C in ambient air. The plates were examined for growth after 24-72 hours. The bacterial isolates were identified employing standard morphological and biochemical procedures such as analytical profile index (API) 20E, API 20NE. The identification of streptococci was confirmed by optodrin disk and bile solubility test. 

#### Antibiotic susceptibility testing

Disk diffusion method was used to determine antimicrobial susceptibility and it predicts the susceptibility based on break-points that correlate zones of inhibition with the minimum inhibitory concentrations (MIC) according to Clinical Laboratory Standards Institute recommendations. Briefly: two to three discrete colonies from an 18-24 hour old cultures were transferred using a loop into a 4-5 ml of sterile normal saline in a tube and emulsified and turbidity of suspension adjusted to match with 0.5 McFarland standards by adding more sterile saline. The isolates were then inoculated in labelled well dried Muller Hinton agar plates using sterile cotton swabs. Appropriate five disks of antimicrobial agents for various organisms were placed firmly on each 100 mm plate and incubated inverted in an incubator at 35°C within 15 minutes. Susceptibility testing were performed on the following antibiotics from Biolab Hungary: ampicillin (AMP), Chloramphenicol (CAF), Ciprofloxacin (CIP), Erythromycin (ERY), gentamycin (GEN), Sulfamethoxazone–trmethoprim (SXT), ceftriaxone, cefuroxime, tetracycline and others, The agar plates were read after 16-18 hours of incubation and results interpreted as follows: the diameters of the zones of inhibition were measured and using the Clinical Laboratory Standards Institute recommended breakpoints (version: Performance of Standards for Antimicrobial Susceptibility Testing; Sixteenth Information Supplement, CLSI 2006) and quality controlled procedures, antibiotic susceptibility were reported as the organism is susceptible, intermediate or resistant. Qualified and certified laboratory personnel with good laboratory practice performed these microbiologic procedures at the department of Medical Microbiology, College of Health Sciences, Makerere University Kampala.

### Data management and analysis

Data collected was double entered using Epi-Data version 3.1 software and exported to STATA version 10.0 (STATA Corp, College station, TX, USA) software for statistical analysis. Univariate analyses were run to determine frequencies, percentages means and median. To determine the proportion of bacterial and fungal co-morbidities isolated from the induced sputum samples, the number of study patients found to have bacteria or fungi was calculated as a percentage of cases among the total number of study population with their corresponding 95% confidence intervals. The distribution of micro-bacterial isolates and antimicrobial susceptibility profiles were reported as proportions, with their corresponding 95% confidence intervals, of the various pathogens that were susceptible to each specific anti-microbial agent. Kappa statistics was used to compare the degree of agreement between PCR and Giemsa methods to detect *P. jirovecii*

### Ethics Statements

The study was approved by Makerere University Medical School Ethics Committee and Uganda National Council for Science and Technology (UNCST). All participants were informed orally in local language (Luganda) or English and written informed consent in Luganda and English obtained. The participants were free to withdraw from the study at any time which would not affect their clinical or health care. Pre-test and post-test HIV counseling were offered and HIV-infected individuals were referred to Mulago Hospital HIV clinic or HIV clinic of their choice for care.

## Results

**Figure 1:**
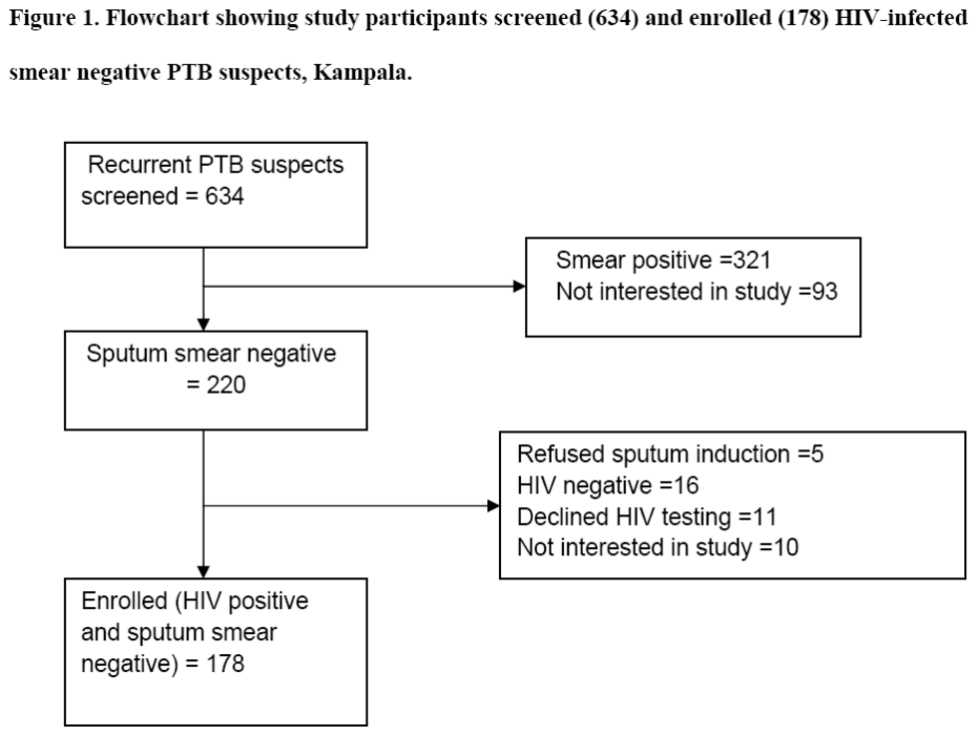
Flowchart showing study participants screened (634) and enrolled (178) HIV-infected smear negative recurrent PTB suspects, Kampala.

Six hundred and thirty four consecutive patients suspected recurrent pulmonary TB were screened and one hundred and seventy eight were enrolled in the study (Figure 1). Fifty four per cent (96/178) of study population were males, median age 37 years (IQR 31-44) and body mass index (BMI) of 19.3 kg/m^2^ (IQR 21.4-17.1). Majority of study participants had low level of education: 82% (146/178) of study participants attained only primary education and 3.4% (6/178) had no formal education. Out of 178 patients, 59 (33.2%) were smokers. High number of participants, 86.5% (154/178) were taking co-trimoxazole compared with only 47.2% on HAART (Table 2). The median CD4 count was 260.5/µL (IQR 106-413). Bacterial, *M. tuberculosis*, and *Pneumocystis jirovecii* pathogens were detected in 48 (27%), 33 (18.5%) and 12 (6.7%) patients, respectively (Table 3). No pathogens were isolated in 53.4% (95/178) of participants. Of the 145 participants culture negative for *M. tuberculosis*, 28.3% (41/145) had bacteria, 7.6% (11/145) were infected with *P. jirovecii* and 6 were co-infected with bacteria and *P. jirovecii*. However among the 33 participants who had *M. tuberculosis*, 7 and 1 were co-infected with bacteria and *P. jirovecii* respectively and none was infected with *M. tuberculosis*, bacteria or *P. jirovecii*. Assessment of the cellular immune status showed that one hundred and four (58.4%) participants had CD4 count of ≥200cells/µL. 

**Table 2:** Socio-demographic and clinical characteristics of study participants of HIV-infected smear negative recurrent PTB suspects Kampala.

**Characteristics**	**Number (N=178)**	**Proportions (%) Participants**
Sex: Male	96	54
Marital status		
Never married	44	24.7
Ever married	134	75.3
Education level		
No formal education	6	3.4
Primary	146	82.0
Post primary	26	14.6
CD4 cell count, cells/µL		
< 200	74	41.1
≥ 200-349	46	25.6
≥ 350	57	31.7
HAART	84	47.2
Co-trimoxazole prophylaxis	156	86.7
Fever		
≤37°C	126	70.8
>37°C	52	29.2
Symptoms		
Purulent sputum	129	72.5
Chest pain	104	58.4
Haemoptysis	32	18
Dyspnoea on exertion	133	74.7
Respiratory rate/min		
≤30	150	84.3
>30	28	15.7

About 41% of study participants were at risk of *P. jirovecii* infection and over 80% of them were on co-trimoxazole prophylaxis. Majority of patients presented with purulent sputum and dyspnoea and 18% presented with haemoptysis.

**Table 3:** Pathogens isolated from induced sputum (N= 178) of HIV-infected smear negative suspected recurrent PTB, Kampala.

**Organism**	**No. Isolates (%)**
*M. tuberculosis (culture*)	33 (18.5)
Bacterial pathogens	48(27)
*Pneumocystis jirovecii (PCR*)	12 (6.7)
No pathogens	95 (53.4)
**Total**	**178 (100)**

At least 81.5% of study participants did not have bacteriologically-confirmed *M. tuberculosis* and 53.4% did not have any microorganisms.

*S. pneumoniae* 20.8 (10/48), *M. catarrhalis* 16.7% (8/48) and *H. influenzae* 16.7 % (8/48) were the most frequently isolated microorganisms. Other bacteria detected in this study include: *E.coli* (6/48), *Pseudomonas aeroginosa* (5/48), *K. pneumoniae* (4/48), S. pyogenes (3/48), *S. aureus* (2/48) and 1 each of *C. feundi* and *P. mirabilis* (Table 4). Most of the microorganisms isolates were susceptible to chloramphenicol, ciprofloxacin and erythromycin. The bacterial susceptibility to chloramphenicol were as follows*: S. pneumoniae* 100%, *M. catarrahalis* 86%, H. influenzae 100%; to ciprofloxacin were: *S. pneumoniae* 100%, *M. catarrahalis 100%, H. influenzae* 100%; to erythomycin were *S. pneumoniae* 100%, *M. catarrahalis* 33%. However, all bacterial strains exhibited resistance to co-trimoxazole except *S. pneumoniae* which was 10% susceptible (Table 5).

**Table 4:** Bacterial isolates from induced sputum of HIV-infected smear negative recurrent PTB suspects, Kampala.

**Pathogens isolates**	**No of bacterial isolates**	**Percent**
*Streptoccocus pneumoniae*	10	20.8
*Moraxella catarrhalis*	8	16.7
*Haemophilus influenzae*	8	16.7
*Escherica coli*	6	12.5
*Pseudomonas aeruginosa*	5	10.4
*Klebsiella pneumoniae*	4	8.3
*Streptoccocus pyogenes*	3	6.2
*Staphyloccocus aureus*	2	4.2
*Proteus mirabilis*	1	2.1
*Citrobactor freundii*	1	2.1
**Total**	**48**	**100**

*S. pneumoniae, M. catarrhalis, H. influenzae* and *E. coli* were the dominant bacteria isolated from induced sputum samples of study participants.

**Table 5:** Micro-organisms isolates from induced sputum and antibiotic susceptibility profiles of HIV positive smear negative recurrent PTB suspects.

**Pathogen: no of isolates (%)**	**Agent**	**Resistant **		**Intermediate **		**Sensitive **	
		**n**	**%**	**n**	**%**	**n**	**%**	
***Streptococcus pneumoniae***	Chloramphenicol	0	0	0	0	10	100	
10(20.8)	Co-trimoxazole	9	90	0	0	1	10	
	Erythromycin	0	0	0	0	10	100	
	Penicillin	9	90	0	0	1	10	
***Moraxella catarrhalis***	Ampicillin	3	60	1	20	1	20	
8(16.7)	Augmentin	1	17	1	17	4	67	
	Ceftriaxone	0	0	0	0	1	100	
	Chloramphenicol	1	14	0	0	6	86	
	Ciprofloxacin	0	0	0	0	8	100	
	Co-trimoxazole	8	100	0	0	0	0	
	Erythromycin	2	67	0	0	1	33	
	Gentamycin	0	0	0	0	4	100	
	Penicillin	0	0	0	0	1	100	
	Tetracycline	1	100	0	0	0	0	
***Haemophilus influenzae***	Ampicillin	2	25	0	0	6	75	
8(16.7)	Augmentin	2	25	0	0	6	75	
	Chloramphenicol	0	0	0	0	8	100	
	Co-trimoxazole	8	100	0	0	0	0	
***Escherichia coli***	Amikacin	0	0	0	0	1	100	
6(12.5)	Ampicillin	6	100	0	0	0	0	
	Augmentin	2	33	1	17	3	50	
	Ceftriaxone	0	0	0	0	5	100	
	Cefuroxime	1	50	0	0	1	50	
	Chloramphenicol	0	0	1	17	5	83	
	Ciprofloxacin	3	50	0	0	3	50	
	Co-trimoxazole	6	100	0	0	0	0	
	Gentamycin	1	17	0	0	5	83	
	Imipenem	0	0	0	0	2	100	
	Meropenem	0	0	0	0	1	100	
***Pseudomonas aeruginosa***	Amikacin	0	0	0	0	5	100	
*5(10.4)*	Aztreonam	0	0	0	0	1	100	
	Ceftazindime	1	20	1	20	3	60	
	Ciprofloxacin	1	20	0	0	4	80	
	Gentamycin	0	0	0	0	4	100	
	Piperacillin	1	20	0	0	4	80	
	Tetracycline	1	100	0	0	0	0	
**Other 11(22.9)**	Amikacin	0	0	0	0	1	100	
*Klebsiella pneumoniae 4(8.3)*	Ampicillin	6	100	0	0	0	0	
*Streptococcus pyogenes 3(6.2)*	Augmentin	3	75	0	0	1	25	
*Staphylococus aureus 2(4.2*)	Cefotaxime	0	0	0	0	1	100	
*Citrobacter freundii 1(2.1)*	Ceftazindime	2	67	0	0	1	33	
*Proteus mirabilis 1(2.1)*	Ceftriaxone	4	80	0	0	1	20	
	Cefuroxime	1	100	0	0	0	0	
	Chloramphenicol	3	38	0	0	5	63	
	Ciprofloxacin	2	22	0	0	7	78	
	Co-trimoxazole	9	100	0	0	0	0	
	Erythromycin	1	25	0	0	3	75	
	Gentamycin	3	38	0	0	5	63	
	Imipenem	0	0	0	0	3	100	
	Oxacillin	0	0	0	0	2	100	
	Penicillin	2	67	0	0	1	33	
	Tetracycline	1	50	0	0	1	50	
	Vancomycin	0	0	0	0	2	100	

Concentration of antimicrobioal agents: AMP, GEN 10µg/ml, CIP 5µg/ML, SXT 23.75/1.25µg/ml Sulpharmethoxazole / Trimethoprim, CEF, ZOX, CHL 30µg/ml.

AMP=Ampicillin, GEN=Gentamycin, CIP=Ciprofloxacin, SXT=Sulpharmethoxazole/Trimethoprim,CHL=Chloramphenicol, ZOX=Cetrizoxime, CEF=Ceftriaxone

Of the 48 patients who had bacterial isolates, 9 (19%), 3 (1%) had radiological evidence of cavities and pleural effusion respectively. However, of the 33 patients who had *M. tuberculosis*, 7 (2%) had cavities, 1 miliary pattern and no pleural effusion. There was one chest x-ray showing dilated bronchioles (honey combing) typical of bronchiectasis in a patient who had no microorganisms isolated from his sputum sample.

## Discussion

Our study findings have shown that bacteria were the commonest microorganisms isolated among 145 participants who had negative cultures for *M. tuberculosis*. Of the 33 participants with culture confirmed *M. tuberculosis*, 7 and 1 had co-morbidity with bacteria and *P. jirovecii* respectively. *Streptococcus pneumoniae*, *Moraxella catarrhalis* and *Haemophilus pneumoniae* were the most frequent microorganisms isolated and most of these microorganisms were susceptible to chloramphenicol, ciprofloxacin and erythromycin but were frequently resistant to co-trimoxazole.

### *M. tuberculosis*

The prevalence of *M. tuberculosis* in smear negative HIV-infected recurrent PTB suspects has not been known previously in Uganda. In this study, *M. tuberculosis* was isolated in only 18.5% of smear negative HIV-infected participants who presented with pulmonary symptoms being evaluated for recurrent tuberculosis. This finding is lower than 42% of 60 smear negative PTB patients who had previously been treated in Malawi [14]. Other studies in the sub-Saharan Africa that used bronchoscopy to obtain broncho-alveolar lavage fluid, have found *M. tuberculosis* prevalence rate of 10-24.1% [7,8,10,12,13]. In resource limited settings where culture technology for *M. tuberculosis* is limited to only one or few reference laboratories, most of the sputum smear negative PTB recurrent suspects would be started on re-treatment regimen before confirmation, in this case 81.5%. This is not cost effective, and it exposes patients on HAART and co-trimoxazole to high pill burden which may reduce adherence, increase the risks of drug-drug interactions and overlapping drug toxicities [23,24,25,26]. Concurrent therapy of TB-HIV co-infection requires concomitant administration of four different anti-tuberculosis drugs for three months supplemented with streptomycin for the first two months of intensive phase followed by three drugs for five months in the continuation phase as well as three antiretroviral agents. The toxicities of some anti-retroviral drugs may overlap with or be additive to toxicities due to anti-tuberculosis medications [27]. The current standard of care for the treatment of HIV infection is triple therapy with two nucleoside reverse transcriptase inhibitor (NRTI) or nucleotide reverse transcriptase inhibitor (NtRTI) backbones, for example, lamivudine and zidovudine in combination with a non-nucleoside reverse transcriptase inhibitor (NNRTI) (efavirenz and nevirapin) or a protease inhibitor (PI) [28,29]. The multiple drug toxicities and the pharmacokinetic interactions between the PIs and NNRTIs and the rifamycins, key components of combination therapy for HIV and TB disease respectively, severely limit the options for optimal HAART regimens during rifampicin based TB therapy [27]. The interactions between the rifamycins and the NNRTs and the PIs are complex. The PIs and NNRTs are metabolized mainly through the cytochrome P450 (CYP) 3A4 enzymes. The rifamycins induce the expression of CYP3A4 isoenzyme in the liver and intestines [30,31], thereby greatly reducing the plasma concentration and exposure to the PIs and the NNRTIs when administered together [23]. In addition rifampicin increases the activity of efflux multidrug transporter P-glycoprotein (P-gp), which contributes to the elimination of the PIs. The reduction in plasma concentration of PIs and the NNRTIs during concurrent treatment with rifampicin can be associated with HIV treatment failure and emergence of drug resistance. In this study, 14/33 participants who had *M. tuberculosis* were on HAART and it is probable that they were failing the HAART therapy. Of the culture positive for *M. tuberculosis*, 3% (1/33) study participants had multiple drug resistant TB (MDR-TB), a condition where the *M. tuberclosis* is resistant to at least rifampicin and isoniazid anti-tuberculosis medications. It is known that HIV-infected individuals who have MDR-TB have very high mortality [32]. In this clinic setting, MDR-TB strains can easily be transmitted to many contacts who are HIV-infected. The study in rural area of KwaZulu Natal showed that drug resistant TB was transmitted to HIV-infected patients and was associated with a high mortality of 98% [32]. It is therefore prudent that rapid detection technology, such as line probe assay (LPA), of MDR-TB cases should be put in place. Rapid detection of drug resistant cases will improve on effective triage mechanism and improve on the infection control measures in a TB and TB/HIV clinic setting in general. Transmissions of drug resistant *M. tuberculosis* strains to health care workers and TB patients can therefore be reduced significantly this way. 

### Bacteria

*S. pneumoniae*, *M. catarrhalis* and *H. influenzae* were the most frequently isolated microorganisms (Table 4). *S. pneumoniae* and *H. influenziae* are common causes of chest infection in HIV-infected adult patients [4,33]. Some studies conducted in Africa have shown that *Streptococcus pneumoniae* is the commonest pathogen that occur in HIV-infected individuals [7,34,35]. Other pathogens detected in this study include: *E. coli*, *P. aeruginosa*, *K. pneumoniae*, *P. mirabilis*, *S.* pyogenes, *C. feundi* and *S. aureus*. The bacterial infections are common in this study population because HIV is also associated with substantial dysregulation of humoral immunity, since CD4 lymphocytes regulate B cell differentiation and indirectly affect antibody production and phagocytosis [4]. 

In this study, *S. pneumoniae* isolates showed both frequent and low rates of susceptibilities to first line antibiotics tested. There was 90% resistance to co-trimoxazole and this finding is consistent with studies reported from the sub-Saharan Africa [36,37]. *S. pneumoniae* also exhibited 90% resistance to penicillin, similar to pattern of resistance found in studies done in this region [36,38,39]. However, our study showed no resistance of *S. pneumoniae* to chloramphenicol compared to 33% resistance in Malawi [37]. In our study, most of the bacterial pathogens were susceptible to chloramphenicol, ciprofloxacin and erythromycin but were resistant to co-trimoxazole (Table 5), an antibiotic that is widely used as a prophylaxis against opportunistic infections in HIV/AIDS infected persons as recommended by WHO [40]. Only *E. coli* and *H. influenzae* were 100% susceptible to co-trimoxazole. Although no information was collected on recent or current antibiotic use by study participants except for co-trimoxazole, poor prescription culture and irrational use of antibiotics in Uganda which worsens the health outcomes associated with pneumonia in resource limited settings [41,42] could be attributable in part, to these antimicrobial resistance patterns.

### *P. jirovecii*

**Figure 2:**
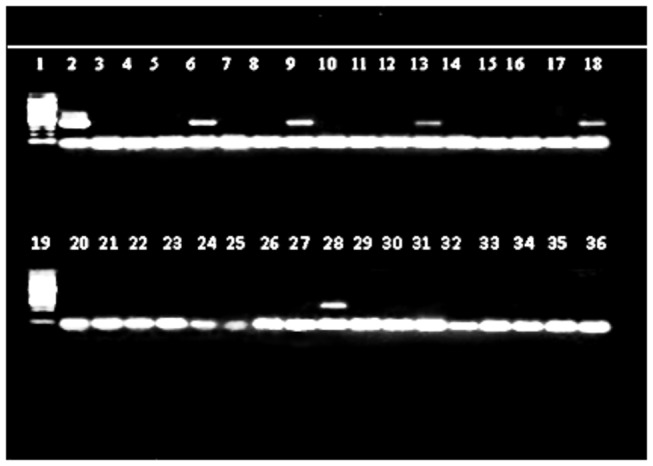
Gel electrophoresis picture for the PCR of the 18S rRNA gene in *P. jirovecii* of HIV-infected smear negative recurrent PTB suspects, Kampala. Note: Lanes 1 and 19 are 100bp DNA ladders. Lane 2 has the positive control DNA. Lanes 6, 9, 13, 18 and 28: *P.jirovecii* positive. Lanes 3,4,5,8, 10, 11, 12, 14, 15, 16, 17, 20, 21, 22, 23, 24, 25, 26, 27, 29, 30, 31, 32, 33, 34, 35 and 36: *P.jirovecii* negative samples.

HIV associated *P. jirovecii* is reported at various rates throughout the world [43,44]. In this study, 6.7% of study participants had *P. jirovecii* isolated from induced sputum samples. This rate of *P. jiorvecii* is lower than those in other previous studies done in Kampala [8,10]. This observed difference could be because of the following possible reasons: different populations studied, use of co-trimoxazole and other antibiotics, use of HAART by study populations, the methods employed to detect *P. jirovecii* organisms and the specimens examined for *P. jirovecii*. The study population in other studies consisted of smear negative for AFB individuals being evaluated for acute pneumonia [7,8,10,12]. Clinical studies from Africa that performed bronchoscopy with bronchoalveolar lavage in HIV-infected patients with pneumonia report that *P. jirovecii* accounted for 0.8-38.6% of cases [8,35,44]. In our study, 86% of study participants were on co-trimoxazole prophylaxis for at least three weeks. The use of co-trimoxazole might have reduced the number of *P. jirovecii* organisms hence contributed to low prevalence of *P. jirovecii* colonization and/or infection and also micro-organisms isolated. To our knowledge, this is the only study that has been conducted and evaluated both CD4 T cell counts and the use of HAART by study participants. The use of HAART with good adherence improves recovery of immune system, reduces incidence of opportunistic infections [45,46] and reduces mortality [24,47,48]. In this study, 48 % of participants were on HAART. Polymerase chain reaction (PCR) technique to detect 18S rRNA gene of *P jirovecii*, was used as previously described [49] and has a sensitivity of 100% but reduced specificity. The 18S rRNA gene PCR nonspecifically amplified *C. cerevisiae* and *C. albicans* [50]. However, previous other studies used immunoflorescent and or Giemsa stains. PCR is more sensitive [51,52,53,54,55,56] and specific [49] than both immunoflorescence and Giemsa techniques used in other above reported studies. Our study participants tested 6.7 % and 12% positive by PCR (Figure 2 but 7 positive lanes not shown) and Giemsa methods respectively and there was concordance in only three positive results. This implies poor strength of agreement (Kappa=0.1050, p=0.071). The conflicting results of the two tests could be influenced by use of co-trimoxazole prophylaxis [57] and 11 of the 12 patients who had *P. jirovecii* were on co-trimoxazole for a minimum duration of three weeks.

Although viral causes of pulmonary symptoms were not investigated in this study, they are common pathogens in HIV-infected individuals. A study in Moshi, Tanzania found that 10% of study population identified HHV8 in BAL fluid as the causative agent of pulmonary symptoms in HIV-infected patients [7].

### Correlating level of immunity and distribution of micro-organisms

In our study, the overall median CD4 T cell count was 260.5 cells/µL (IQR 106-413). This median CD4 T cell count implies low level of immunity below the average normal range of 410-1800 cells/µL. However the median CD4 T cell counts in participants whom micro-organisms were detected was 286 cells/µL (IQR 99-413) and those without micro-organisms was 250 cells/µL (IQR 106-419), (Pearson chi^2^ test, p=0.45). This difference was not statistically significant. The higher level of CD4 T cells count in participants infected with microorganisms than in participants without is surprising but could be explained in part, by more use of antibiotics (41/48) by participants not infected by microorganisms. Also immunological assessment of participants infected with *M. tuberculosis* found a median CD4 T cells count of 262 cells/µL (IQR 93-419); participants with bacteria 268 cells/µL (IQR 103.5-362.5) and participants with *P. jirovecii* 284.5 cells/µL (IQR 110-429). The Pearson chi ^2^ tests showed no statistically significant difference in CD4 T cell count between these groups (Table 6). These observed findings could be attributed to the use of HAART which inhibits HIV viral replication and allows for CD4 T cell related immune reconstitution that could have protected the participants against opportunistic infections at all stages of HIV disease [24,47,48,58].

**Table 6:** Correlation between aetiologic pathogens isolates from induced sputum samples and CD4 T cell counts of smear negative HIV-infected PTB suspects, Kampala.

Pathogens isolated from induced sputum	Number of pathogens isolated	Median CD4 T cell count/µL (IQR)	Pearson chi^2^ test (p-value)
*M. tuberculosis*	33	262 (419-93)	0.85
Bacteria	48	268 (362.5-103.5)	1.00
*P. jirovecii*	12	284.5 (429-110)	0.55

There were no associations between CD4 counts and prevalence of *M. tuberculosis*, bacteria and *P. jirovecii* (*P<0.5, Pearson chi* ^*2*^ *test*)*.*

 There were some limitations to the study which included inadequate financial resources. We could not, for example do chest x-ray on all patients. A cross-sectional study also has its limitations compared to prospective longitudinal study in that study outcomes cannot be realized. Viral causes of pulmonary symptoms were also not investigated in this study.

## Conclusion

At least 81.5% of HIV-infected smear negative recurrent TB suspects did not have microbiologically-confirmed TB. However one case of MDR-TB was found and while *M. tuberculosis* was the single most common micro-organism, bacterial pathogens dominated with *S. pneumoniae, Moraxella catarrhalis*, and *H.influenzae* being the most frequent isolates. Almost all the bacteria isolates were resistant to co-trimoxazole except *E, coli* and *H. influenzae*, More than 86% of study participants reported co-trimoxazole prophylaxis use. The prevalence rate of *P. jirovecii* in our study finding is lower than in two previous studies done in Mulago Hospital, possibly because of effective use of co-trimoxazole by the patients. Although 53.4% of patients did not have microorganisms, viral causes were not excluded. Our findings call for a thorough investigation of HIV-infected smear negative recurrent PTB suspects with pulmonary symptoms in resource limited settings to help arrive at the right diagnosis and guide cost effective treatment. 
